# Supplementary material for: Quantum phase transition dynamics in the two-dimensional transverse-field Ising model
Source: Sci Adv. 2022 Sep 16;8(37):eabl6850. doi: 10.1126/sciadv.abl6850 (PMC9481121; doi:10.1126/sciadv.abl6850)
Supplement: Supplementary file 1 — Figs. S1 to S3 [file sciadv.abl6850_sm.pdf]

Science Advances
AAAS

Supplementary Materials for

Quantum phase transition dynamics in the two-dimensional transverse-field Ising model

Markus Schmitt *et al.*

Corresponding author: Markus Schmitt, markus.schmitt@uni-koeln.de; Marek M. Rams, marek.rams@uj.edu.pl

Sci. Adv. **8**, eabl6850 (2022)
DOI: 10.1126/sciadv.abl6850

This PDF file includes:

Figs. S1 to S3

LINEAR VERSUS SMOOTH RAMP

In the main text we consider a straight linear ramp and one that is smoothed at the ends. The straight ramp with its sharp beginning (discontinuous time derivative) is inconvenient for tensor networks (TN) simulations because from the very start it generates some excitations that have nothing to do with the KZ mechanism in question but whose entanglement has to be accounted for by extra bond dimension of the networks. This does not seem to be a problem for the neural network (NN) and that is why the NN employs the linear ramp that also requires less time to simulate. For the same reason it would be more convenient to perform in an experiment.

The precise beginning and ending of the ramp does not affect the KZ mechanism because KZ excitations are set near the critical point which is right in the middle between the beginning and the end. This can be seen in e.g. Fig. 4**A** where there is no appreciable difference between NN and TN results. Small discrepancies between NN and TN in Fig. 4**B** are solely due to their different boundary conditions which are, respectively, periodic and open and, therefore, the discrepancies are finite size effects, compare Fig. S1 below where open boundary conditions are assumed for both the linear and the smooth ramp. This independence on ramp's beginning/ending is very convenient for experimental implementations giving them some flexibility.

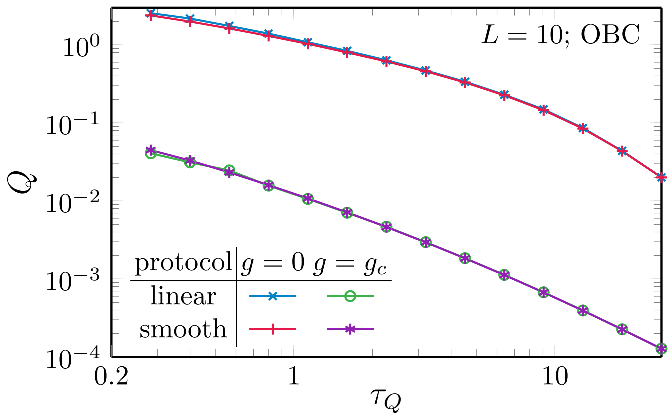

Figure S1. **Independence of the KZ mechanism on the ramp details.** We compare the excitation energy for a linear ramp in Eq. (1) of the main text, and a ramp that is smoothed at the ends as in Eq. (8) of the main text. Both ramps have the same slope – set by τ_Q – when crossing the critical point. We show excitation energy per spin measured at the critical point, and at the end of the ramp when $g = 0$. The details of the ramp have a marginal influence on the resulting excitation energy. There is a very small "cooling" effect related to a slightly longer time for coarsening dynamics in the smooth ramp that is visible for $g = 0$ and fast quenches – consistent with trends in Fig. 7. This is, however, a sub-leading effect.

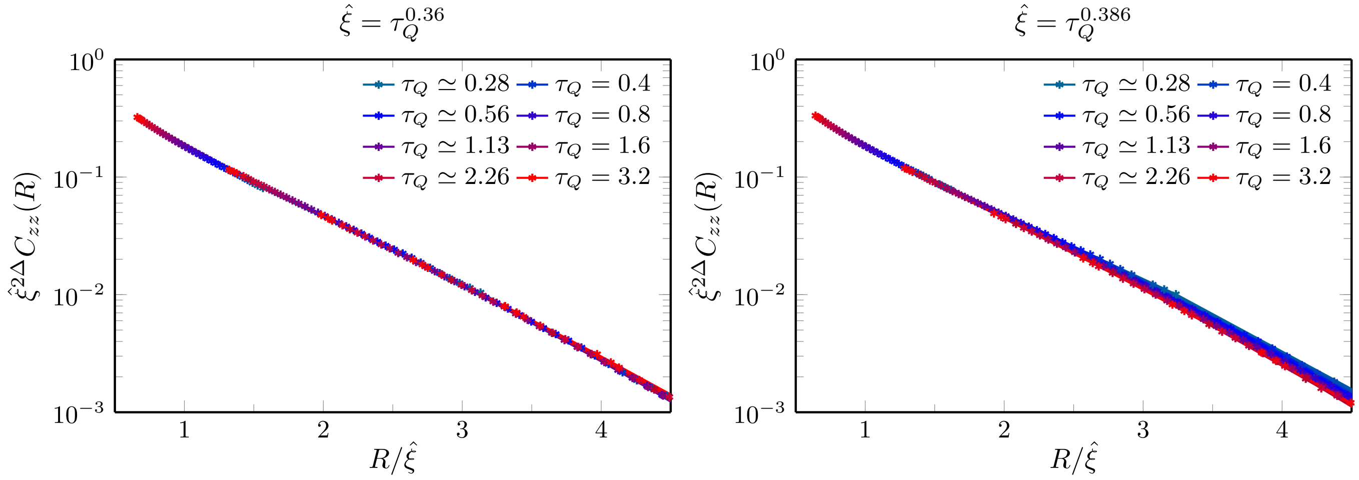

Figure S2. **The best collapse of the correlation function.** Scaled correlation function for 36 values of $\tau_Q = 0.1 \cdot 2^{m/10}$, with $m = 15, \ldots, 50$. In the left panel we assume $\hat{\xi} = \tau_Q^{0.36}$ and in the right one $\hat{\xi} = \tau_Q^{0.386}$. The collapse on the left is clearly better than on the right for available range of quench times τ_Q.

DYNAMICAL EXPONENT

In Fig. S2, we compare collapses of the correlation function obtained by iPEPS on an infinite lattice for the range of quench times $0.28 \leq \tau_Q \leq 3.2$ with the exponents 0.36 and the exact 0.386. Considering the longest τ_Q that we can achieve here, the effective 0.36 clearly yields a better collapse than 0.386. The collapse for 0.386 is still decent, but mainly because the two exponents differ by just 7%. The precision of our numerical data is good enough to discriminate this small difference.

EXTRAPOLATION OF THE ENERGY GAP

The inset in Fig. 2 of the main text tests the finite-size scaling hypothesis for the energy gap near the critical point. The collapse is compelling, especially on the paramagnetic side. In Fig. S3 below, we use the data on the paramagnetic side to extrapolate the gap in system size up to $L = 100$. The extrapolated data agrees with the expected power-law with exponent $z\nu \approx 0.63$, and give an impression of the significance of finite size effects close to the critical point. The extrapolation to $L = 20$, which is the size we use to simulate quenches, is also shown in Fig. 2 of the main text.

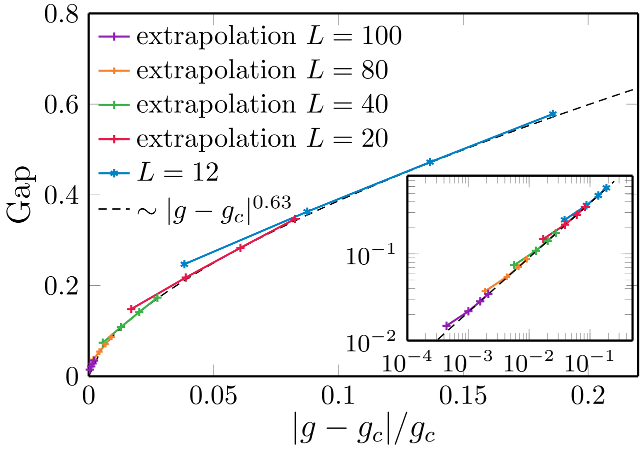

Figure S3. **Extrapolation of the energy gap in larger system sizes.** We extrapolate the energy gap on the paramagnetic side of the critical point for a few large system sizes based on the finite-size scaling collapse shown in the inset of Fig. 2 of the main text.
